# Chemokine CXCL16 Expression Suppresses Migration and Invasiveness and Induces Apoptosis in Breast Cancer Cells

**DOI:** 10.1155/2014/478641

**Published:** 2014-04-22

**Authors:** Yeying Fang, Fraser C. Henderson, Qiong Yi, Qianqian Lei, Yan Li, Nianyong Chen

**Affiliations:** ^1^Department of Radiation Oncology, Cancer Center and State Key Laboratory of Biotherapy, West China Hospital, Sichuan University, 37 Guoxuexiang, Chengdu, Sichuan 610041, China; ^2^University of Virginia School of Medicine, Charlottesville, VA 22908, USA

## Abstract

*Background.* Increasing evidence argues that soluble CXCL16 promotes proliferation, migration, and invasion of cancer cells *in vitro*. However, the role of transmembrane or cellular CXCL16 in cancer remains relatively unknown. In this study, we determine the function of cellular CXCL16 as tumor suppressor in breast cancer cells. *Methods.* Expression of cellular CXCL16 in breast cancer cell lines was determined at both RNA and protein levels. *In vitro* and *in vivo* studies that overexpressed or downregulated CXCL16 were conducted in breast cancer cells. *Results.* We report differential expression of cellular CXCL16 in breast cancer cell lines that was negatively correlated with cell invasiveness and migration. Overexpression of CXCL16 in MDA-MB-231 cells led to a decrease in cell invasion and migration and induced apoptosis of the cells; downregulation of CXCL16 in MCF-7 cells increased cell migration and invasiveness. Consistent with the *in vitro* data, CXCL16 overexpression inhibited tumorigenesis *in vivo*. *Conclusions.* Cellular CXCL16 suppresses invasion and metastasis of breast cancer cells *in vitro* and inhibits tumorigenesis *in vivo*. Targeting of cellular CXCL16 expression is a potential therapeutic strategy for breast cancer.

## 1. Introduction

CXCL16, a newly identified chemokine, has been described in both transmembrane and soluble forms [1]. The orphan receptor for CXCL16 is CXCR6 [2]. Transmembrane CXCL16 (TM-CXCL16) is expressed on the surface of macrophages, dendritic cells, and monocytes where it functions as an adhesion molecule for CXCR6-positive immune cells [1, 3]. TM-CXCL16 undergoes cleavage by the disintegrin-like metalloproteinases ADAM10 and ADAM17 before it is released in soluble form to the outside of the cell [4, 5]. Soluble CXCL16 (sCXCL16) prompts migration of leukocytes expressing CXCR6 in a dose-dependent manner [6].

Because cancer cell migration and metastasis share patterns with leukocyte trafficking [7], attention has been focused on the role of CXCL16 in cancer progression. CXCL16 is expressed in various cancers, including pancreatic, prostate, breast, colorectal, and nonsmall cell lung cancer [8, 9]. More importantly, Lu et al. revealed a positive correlation between CXCL16 mRNA expression and prostate cancer aggressiveness such that metastatic lesions expressed higher levels of CXCL16 mRNA than the primary prostate tissues [9]. These results suggest a role for CXCL16 in cancer aggressiveness.

*In vitro* studies have shown that sCXCL16 induces migration and proliferation of CXCR6-expressing prostate cancer cells [9, 10]. Moreover, Matsushita et al. reported that high preoperative serous levels of sCXCL16 were associated with liver recurrence and poor prognosis in patients with colorectal cancer [11]. TM-CXCL16 has been less studied. Immunohistochemical staining data from patients with colorectal or renal cancer correlated better long-term prognosis with stronger CXCL16 staining in cancer tissues [12, 13]. These limited reports imply different functions for CXCL16 depending on the location of its expression in cancer patients.

Breast cancer is the most common malignancy and the second leading cause of cancer-related death in American women. Despite survival rates having improved steadily since 1990, the impact of breast cancer on overall mortality continues to grow [14]. Therefore, it is important to get a better understanding of the molecular mechanisms underlying breast cancer metastasis and to develop prognostic and therapeutic strategies. In this study, we explore the expression and function of CXCL16 in breast cancer cell lines that differ in aggressiveness.

## 2. Materials and Methods

### 2.1. Cell Culture

The breast cancer cell lines SK-BR-3, MCF-7, and MDA-MB-231 were obtained from American Type Culture Collection (ATCC) (Rockville, MD). The noncancerous human mammary epithelial cell line MCF-10A was purchased from Bioleaf Biotech (Shanghai, China). All cell lines were cultured at 37°C in Dulbecco's Modified Eagle Medium (Hyclone, Waltham, MA) supplemented with 10% fetal bovine serum, 100 units/mL penicillin, and 100 ug/mL streptomycin in a humid incubator with 5% CO_2_.

### 2.2. Quantitative RT-PCR

Total RNA was extracted by Biozol reagent (Bioflux, Tokyo, Japan) according to the manufacturer's instructions. Less than 2 ug RNA was reverse-transcripted into cDNA using reverse transcriptase (Promega, Beijing, China) and oligo(dT)18 (Takara, Dalian, China). Primers for CXCL16 were as follow: sense 5′-GGCCCACCAGAAGCATTTAC-3′ and antisense 5′-CTGAAGATGCCCCCTCTGAG-3′. Primers for glyceraldehyde 3-phosphate dehydrogenase were as follows: sense 5′-GAAGGTGAAGGTCGGAGTC-3′and antisense 5′-GAAGATGGTGATGGGATTTC-3′. PCR was performed with an iQ4 Multicolor Real-Time PCR Detection System (Bio Rad, Hercules, CA) using Sso Fast EvaGreen Supermix (Bio Rad). PCR protocol was performed as follows: denaturing for three seconds at 98°C followed by forty amplification cycles of annealing and extension at 55°C for fifteen seconds.

### 2.3. Western Blot

Cells were lysed in ice-cold radioimmunoprecipitation assay (RIPA) buffer. Protein concentration was measured with the Bradford assay. Normalized lysates (30 ug) were separated by electrophoresis in 12% SDS-PAGE and electrotransferred onto polyvinylidene fluoride membrane (PVDF membrane, Millipore, Billerica, MA). The membrane was blocked with 5% nonfat milk in Tris-buffered saline-Tween (TBST, Ph 7.6) at room temperate for 1 h and incubated overnight at 4°C with CXCL16 antibody (Abcam, Cambridge, UK). After three washes with TBST, the membrane was incubated with horseradish peroxidase- (HRP-) conjugated IgG. Signals were visualized with enhance chemiluminescence (ECL; Millipore).

### 2.4. Flow Cytometry

Cells were trypsinized and 10^6^ cells were incubated with PE-conjugated CXCL16 antibody (R&D Systems, Minneapolis, MN) in a dark room for 45 min. After two washings with phosphate buffered solution (PBS), expression of transmembrane CXCL16 in cells was analyzed with a Becton Dickinson FACScan using a software FACS express 3 (De Novo Software, Los Angeles, CA).

### 2.5. Proliferation, Migration, and Invasion Assay

Proliferation was determined by 3-(4,5-dimethylthiazol-2-yl)-2,5-diphenyltetrazolium bromide (MTT) assay. Cells were seeded with a volume of 200 ul (2,000 cells/well) into 96-well plates (Corning). Every 24 h, MTT was added to the well with a final concentration of 0.5 mg/mL and subsequently incubated for 4 h at 37°C. Supernate was discarded and 150 ul/well DMSO was added. The optical densities (OD) were measured at 490 nm with a microplate reader (Bio Rad). The experiment was carried out three times.

Migration and invasion assays were performed using a transwell chamber (8 um pore size, Millipore) according to the manufacturer's instructions. Cell culture inserts for the invasion assay were precoated with Matrigel (BD Biosciences, Bedford, MA) for 4 h at 37°C. Cells were seeded into the upper chamber, while 1 mL complete medium was added into the lower chamber as a chemotaxin. After culture for 24 h, noninvading cells were removed with a cotton bud. Cells that migrated to the lower surface were fixed in 4% paraformaldehyde for 20 min and underwent Giemsa staining. Five random fields were selected for cell counting under a light microscope (100×; Nikon, Tokyo, Japan). The migration assay procedure was similar except that Matrigel was not utilized.

### 2.6. Cell Apoptosis Assay

Caspase-3 was measured using the Caspase-3 Activity Kit (Beyotime, Nanjing, China). Appropriate cells were incubated with 30 ul lysis buffer on ice for 30 min and then subjected to centrifugation at 13,000 rpm for 5 min. Bradford reagent was used to determine the protein concentration. 10 ul of supernatant was incubated with Ac-DEVD-pNA at 37°C for 5 h. The OD values were detected at 405 nm. Annexin V-7 AAD reagent was purchased from Keygentec (Nanjing, China). Cells were collected with EDTA^−^ trypsin and washed twice with phosphate buffer solution (PBS). In a 500 ul binding buffer, cells were stained with 5 ul Annexin V and 5 ul 7 AAD successively in the dark room. Staining intensity was measured by flow cytometry. Apoptosis index is calculated by the percentage of cells with positive for Annexin V and negative for 7-AAD.

### 2.7. Overexpression of CXCL16 with Recombinant CXCL16 Lentivirus

Recombinant CXCL16 lentivirus and control lentivirus were both constructed by Genechem Co., Ltd. (Shanghai, China). The day before transfection, MDA-MB-231 cells were seeded into a six-well plate at a density of 10^5^ cells/well. After transfection in serum-free medium for 12 h, cells were further cultured in complete medium. Overexpression of CXCL16 protein was confirmed by western blot and flow cytometry.

### 2.8. Downregulation of CXCL16 with Short Hairpin RNA (shRNA)

Downregulation of CXCL16 was performed as previously described [15]. Four target sequences for CXCL16 (GenBank accession number: NM_022059) were designed. 5′-GGACCCATGGGTTCAGGAATT-3′ was selected for knocking down 80% of CXCL16 mRNA. Both CXCL16 and control plasmids were obtained from GenePharma Company (Shanghai, China).

### 2.9. Xenograft Experiments

Five-week-old female NOD/SCID mice were purchased from Beijing HFK Bioscience Co., Ltd. (Beijing, China). Cells were trypsinized into single cell suspension, and 3 × 10^6^ cells were injected subcutaneously into the right-lower flank of each mouse. Tumor growth was monitored twice per week beginning with the third week. Tumor volumes were approximated using the formula *V* = [(*π*/6) × *L* × *W* ^2^], where *L* was the longest axis and *W* was the shortest axis. All mice were sacrificed by cervical dislocation, and the tumors were removed and weighed.

### 2.10. Statistical Analysis

All data are expressed as the mean ± standard deviation (SD) and compiled using SPSS 16.0 (SPSS, Chicago, IL). One-way analysis of variance was used to determine statistical and significant differences between control and treated groups. *P* < 0.05 was considered statistically significant.

## 3. Results

### 3.1. *In Vitro* Expression of CXCL16 in Breast Cancer Cells Is Negatively Correlated with Invasiveness and Migration

**Figure 1:**
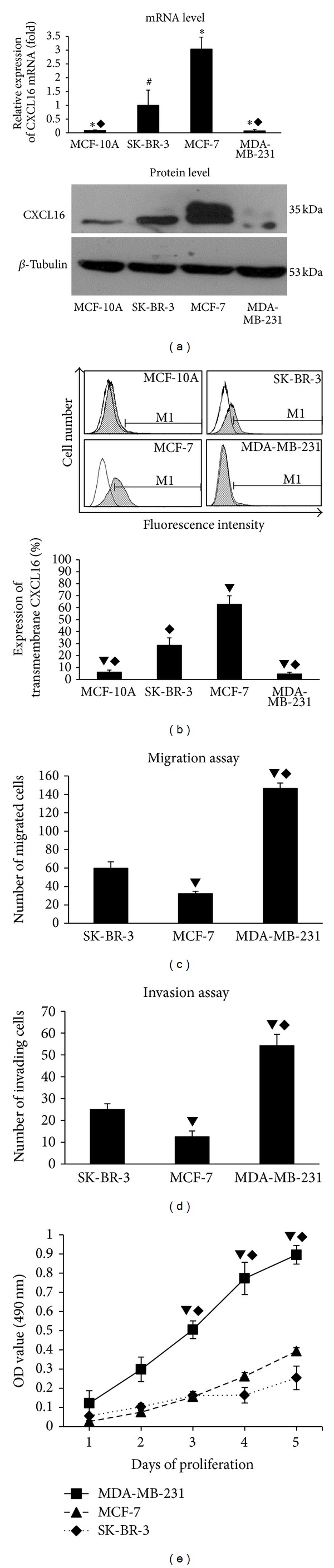
CXCL16 expression in breast cancer cell lines with diverse phenotypic characteristics. (a) CXCL16 mRNA and total protein were highest in MCF-7 and lowest in MDA-MB-231, while noncancerous human mammary epithelial cells MCF-10A faintly expressed CXCL16. *P < 0.05 compared with SK-BR-3. ^◆^ *P* < 0.01 compared with MCF-7. ^#^ *P* < 0.05 compared with MCF-7. (b) TM-CXCL16 expression in breast cancer cell lines. MCF-7 cells expressed the most TM-CXCL16, while MDA-MB-231 cells barely expressed TM-CXCL16. ^▼^ *P* < 0.01 compared with SK-BR-3. ^◆^ *P* < 0.01 compared with MCF-7. (c) and (d) Migratory ability and invasiveness varied by breast cancer cell line. MDA-MB-231 possessed increased migratory ability and invasiveness, while MCF-7 was weakest. ^▼^ *P* < 0.01 compared with SK-BR-3. ^◆^ *P* < 0.01 compared with MCF-7. (e) Proliferation of three breast cancer cell lines. MDA-MB-231 proliferated fastest; no difference was observed between SK-BR-3 and MCF-7. ^▼^ *P* < 0.01 compared with SK-BR-3. ^◆^ *P* < 0.01 compared with MCF-7.

Expression of CXCL16 was evaluated in mammary epithelial cell MCF-10A and in three breast cancer cell lines: SK-BR-3, MCF-7, and MDA-MB-231. As shown in Figure 1(a), CXCL16 mRNA was highest in MCF-7 (3.038 ± 0.436-fold), followed by SK-BR-3 (1 ± 0.548-fold), while the lowest expression was observed in MDA-MB-231 (0.07 ± 0.049-fold). CXCL16 mRNA was markedly lower in noncancerous MCF-10A. Translated CXCL16 as a portion of total protein was similar to that of mRNA. Flow cytometry demonstrated that transmembrane CXCL16 was highest in MCF-7 and lowest in MDA-MB-231 (Figure 1(b)). MCF-7 and SK-BR-3 (with 32.25 ± 2.63 and 59.75 ± 6.94 migrated cells) showed less migration than MDA-MB-231 cells (146.5 ± 5.74, *P* < 0.01) (Figure 1(c)). Invasion assays demonstrated a similar pattern (Figure 1(d)). Proliferation was the strongest in MDA-MB-231 cells, while SK-BR-3 cells and MCF-7 cells possessed less, though equal, proliferative capability (Figure 1(e)).

### 3.2. Overexpression of CXCL16 Suppresses Cell Migration and Invasion and Induces Caspase-3-Dependent Apoptosis

**Figure 2:**
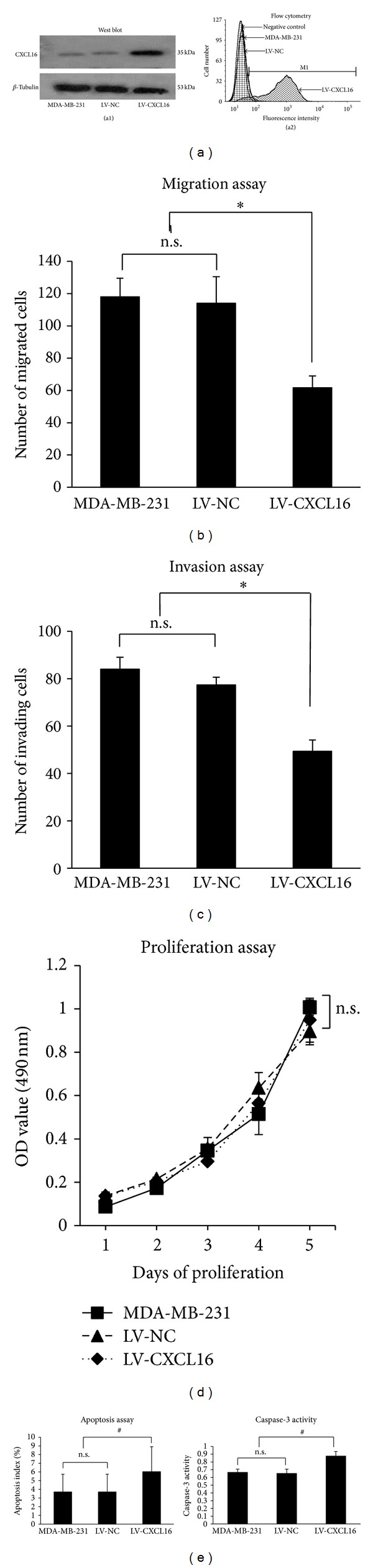
Overexpression of CXCL16 inhibited migration and invasion and promoted apoptosis, of MDA-MB-231 cells. MDA-MB-231 cells were infected with negative lentivirus (LV-NC) or recombinant CXCL16 lentivirus (LV-CXCL16). 120 h later cells were collected and migration, invasion, proliferation, and apoptosis assays were conducted, respectively. (a) Recombinant CXCL16 significantly upregulated total CXCL16 (a1) and TM-CXCL16 (a2). (b) and (c) Overexpression of CXCL16 led to a decrease in migration (b) and invasion (c) of MDA-MB-231 cells. *P < 0.01 compared with MDA-MB-231 or negative control (LV-NC). n.s.: nonsignificant compared between MDA-MB-231 or negative control (LV-NC). (d) Proliferation of MDA-MB-231 cells was not affected by CXCL16 overexpression. n.s.: nonsignificant compared between MDA-MB-231 and negative control (LV-NC). (e) CXCL16 expression promoted apoptosis of MDA-MB-231 cells (left) in conjunction with enhanced caspase-3 activity (right). ^#^ *P* < 0.05 compared with MDA-MB-231 or negative control (LV-NC). n.s.: nonsignificant compared between MDA-MB-231 and negative control (LV-NC).

To further confirm the effect of CXCL16 on invasion, migration, and proliferation in these breast cancer cell lines, MDA-MB-231 cells were stably transfected with recombinant lentivirus CXCL16 plasmid. Elevated CXCL16 levels were confirmed by western blot or flow cytometry (Figure 2(a)). Consistently, CXCL16-overexpressing cells showed significantly decreased cell migration (from 118 ± 11.53 to 61.66 ± 7.37, Figure 2(b)) and invasion (from 84.0 ± 5 to 48.66 ± 4.04, Figure 2(c)) compared with the control mock-transfected cells. However, MTT assay for cell proliferation revealed no difference between cells transfected with CXCL16 plasmids compared with the control mock-transfected cells (Figure 2(d)). In addition, apoptotic index was increased from 3.5 ± 2.03% to 5.9 ± 2.87%, and the activity of caspase-3, a crucial enzyme in the apoptotic cascade, whose OD value was enhanced from 0.65 ± 0.032 to 0.89 ± 0.121, indicated that increased CXCL16 expression facilitated caspase-3-dependent apoptosis (Figure 2(e)).

### 3.3. *In Vitro *Downregulation of CXCL16 in Breast Cancer Cells Increased Invasion and Migration

**Figure 3:**
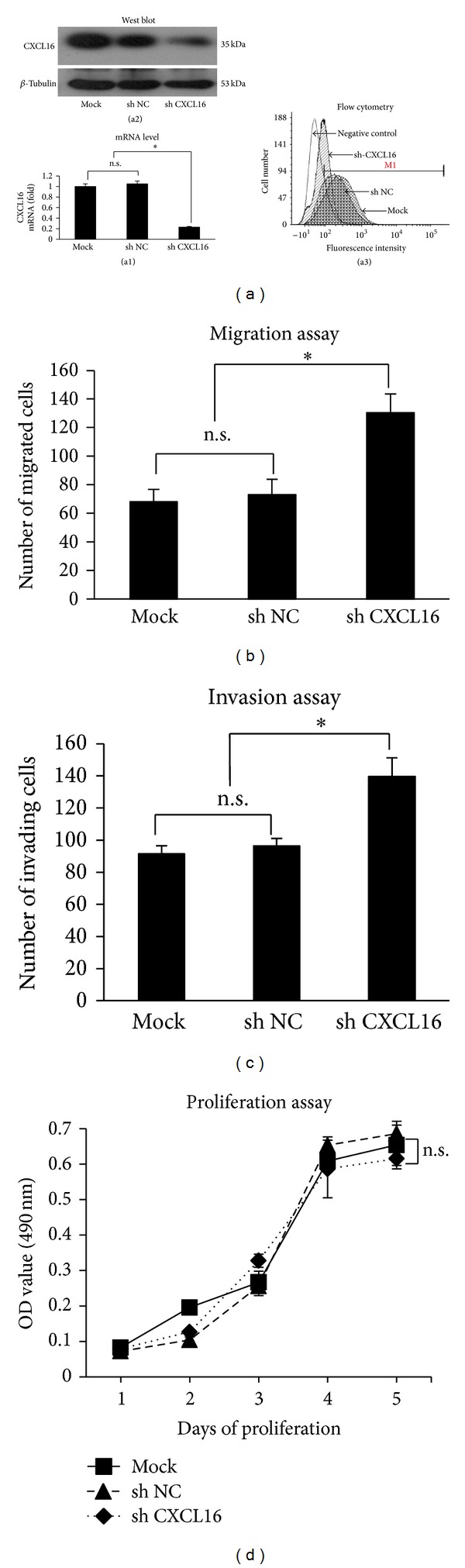
Knockdown of CXCL16 increases migration and invasion, but not proliferation, of MCF-7 cells. MCF-7 cells were treated with lipofectamine alone (mock) or infected with negative plasmids (sh NC) or CXCL16 targeted sequence (Sh CXCL16). 48 h later cells were collected and underwent migration, invasion, and proliferation assays. (a) ShRNA effectively inhibited expression of CXCL16 mRNA ((a1), qRT-PCR), total CXCL16 protein ((a2), western blotting), and TM-CXCL16 ((a3), flow cytometry). (b) CXCL16 knockdown enhanced migration of MCF-7 cells. *P < 0.01 compared with mock or negative control. n.s.: nonsignificant compared between mock and negative control. (c) CXCL16 knockdown enhanced invasion of MCF-7 cells. *P < 0.01 compared with mock or negative control. n.s.: nonsignificant compared between mock and negative control. (d) CXCL16 knockdown had no influence on proliferation of MCF-7 cells. n.s.: nonsignificant compared between mock and negative control (sh NC).

Meanwhile, to determine whether CXCL16 gene silencing would affect breast cancer progression, MCF-7 cells highly expressive for CXCL16 underwent stable knockdown of CXCL16 using shCXCL16, scrambled shRNA (shNC), or liposomes only (mock). The efficiency of CXCL16 knockdown was confirmed by qRT-PCR, western blot, or flow cytometry which showed decreased CXCL16 expression in shCXCL16-transfected MCF-7 cells (Figure 3(a)). Stable downregulation of CXCL16 expression in MCF-7 cells resulted in a significant increase in migration and invasion (Figures 3(b) and 3(c)), but not proliferation (Figure 3(d)).

### 3.4. Increased CXCL16 Expression in Breast Cancer Cells Inhibits Tumorigenesis* In Vivo*

**Figure 4:**
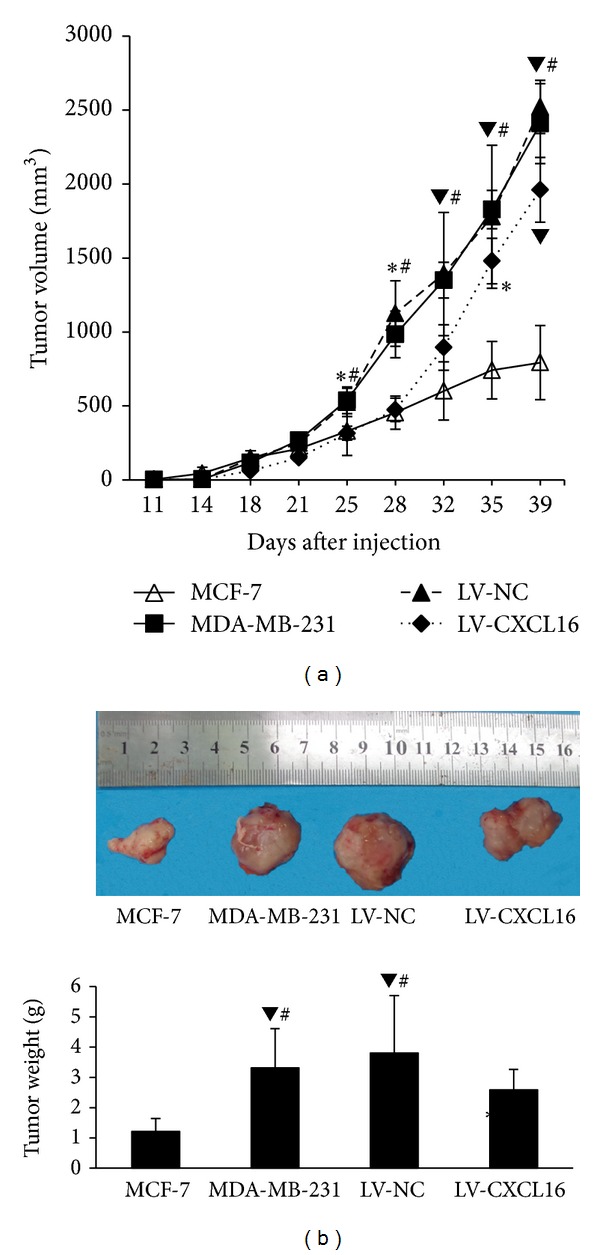
CXCL16 expression inhibited tumor development* in vivo*. Four groups of single cells (MDA-MB-231, LV-NC, LV-CXCL16, and MCF-7) were injected subcutaneously into mice and tumor growth was monitored. (a) CXCL16 overexpression led to a decrease in tumorigenesis. *P < 0.05 compared with MCF-7. ^#^ *P* < 0.05 compared with MDA-MB-231. ^▼^ *P* < 0.01 compared with MCF-7. (b) CXCL16 overexpression reduced tumor volume and weight. ^#^ *P* < 0.05 compared with MDA-MB-231. ^▼^ *P* < 0.01 compared with MCF-7. *P < 0.05 compared with MCF7.

As shown in Figure 4(a), CXCL16-overexpressing MDA-MB-231 showed delayed tumor progression as compared to MDA-MB-231 or the positive control (MCF-7) (*P* < 0.05). Tumor volumes and weights for CXCL16-overexpressing groups were accordingly found to be significantly reduced (*P* < 0.05) (Figure 4(b)).

## 4. Discussion

CXCL16 is a novel chemokine first cloned by Maltoulin in 2000 [2]. Like other chemokines, CXC16 was investigated for its role in immunity. Existing both in soluble form and as a transmembrane form, CXCL16 possessed functions seemingly more nuanced than other well-described chemokines. In soluble form it induces immunocyte chemotaxis, while in transmembrane form CXCL16 mediates cell-cell adhesion [1, 3, 4]. Previous findings from* in vitro* studies using exogenous sCXCL16 suggested that sCXCL16 promotes cell proliferation and invasion. Although Matsushita et al. associated high preoperative levels of sCXCL16 with liver recurrence and poor prognosis in colorectal cancer patients [11], little was known about the functions of TM-CXCL16 except that high expression of CXCL16 in cancer tissues correlated with favorable prognosis in renal and colorectal cancer patients [12, 13]. These observed differences between TM-CXCL16 and sCXCL16 indicate a complicated function for CXCL16 in cancer. We therefore sought to identify a specific function for TM-CXCL16 through* in vivo* and* in vitro* breast cancer models.

In this study, we show for the first time that upregulation of CXCL16 suppresses migration and invasiveness of breast cancer cells* in vitro* and delays progression of tumor growth* in vivo*. These results reveal a protective function of CXCL16 in breast carcinogenesis and present valuable clues to better understanding of the mechanisms of breast cancer progression. Our measurements of both protein and mRNA levels of CXCL16 reveal high expression in MCF-7 cell line, which are positive for estrogen receptor (ER) and progesterone receptor (PR) and therefore considered less aggressive; levels were lower in the receptor triple-negative cancer cell line of MDA-MB-231 which is considered more highly aggressive. This inverse correlation between CXCL16 expression and migration or invasion suggested that CXCL16 play another role as tumor suppressor in inhibiting the migration and invasiveness of breast cancer cells. As discussed above, TM-CXCL16 plays a pivotal role as a tumor suppressor on the development of breast cancer cells; that is, the high expression of CXCL16 or overexpression of CXCL16 significantly suppressed the invasion and migration and induced apoptosis of breast cancer cells; in contrast, low expression of CXCL16 or downregulation of CXCL16 promoted the invasion and migration of breast cancer cells. These findings demonstrated that CXCL16 was directly involved in the migration and invasiveness. Supportively, the studies have reported that the reduced cell-cell adhesiveness is considered one morphological hallmark of malignant tumors [16], and homotypic adhesion was described to reduce the invasive potential of tumor cells [17]. Consistent with previous reports [18], we found that CXCL16 and CXCR6 were coexpressed in breast cancer cells but to inverse extent. When expressed as transmembrane protein, CXCL16 could directly promote cell-cell adhesion by combining with CXCR6 on cell surface [19]. Thus, we speculate here that transmembrane CXCL16 facilitates the cell-cell adhesion like other well described cell adhesion molecules, such as E-cadherin [20], which subsequently prevent the detachment of individual tumor cells from the tumor aggregation and ultimately inhibit cell migration and invasion. CXCL16-mediated cell-cell adhesion may also be regulated by other molecules. Heparin-like glycosaminoglycans are long unbranched polysaccharides consisting of a repeating disaccharide unit that expressed on proteoglycan components of cell surface and extracellular matrix [21]. Cell surface glycosaminoglycans could bind positively charged regions on the protein and lead to enhanced local concentrations of chemokines [22]. For example, heparin binds to the strong positive potential on CXCL12 and stabilizes the CXCL12 dimer on cell surface [23]. Moreover, the presence of cell surface GAGs could enhance the activity of chemokines including MIP-1a, RANTES, or MIP-1b by sequestrating them onto the cell surface. There also has been evidence that GAG is important for CXCL16 recognition and that CXCL16 was able to react with heparin microarrays containing oligosaccharides [24]. However, it remains unknown what the effect of GAG-CXCL16 interactions is and how heparin-like glycosaminoglycan affects the activity of CXCL16.

Another significant finding was that overexpression of CXC16 enhanced apoptosis in MDA-MB-231 in conjunction with upregulation of caspase-3, a crucial enzyme in endogenous apoptosis, suggesting CXCL16 promotes caspase-3-dependent apoptosis. Aberrant balance between proliferation and apoptosis is clearly implicated as one major characteristic in tumor development [25]. However, in this study the apoptosis index or caspase-3 activity in MCF-7 cells is not determined, because the MCF-7 cell line was considered not to express the enzyme caspase-3 [26].

We discovered no role for CXCL16 in the proliferation of breast cancer cell lines in our study, unlike other tumor suppressor studies [27]. It has been speculated that tumor cell lines are normally transformed and have multiple genetic alterations, which may contribute to the noneffect of CXCL16 on the breast cancer proliferation. EGFR overexpression is well-studied in breast cancer [28], and EGFR-driven signaling pathways such as PI3K/AKT/mTOR, JAK/STAT, and Ras/Raf/MAPK were associated with cell proliferation and survival [29]. Since EGFR overexpression is common in breast cancer, the impact of CXCL16 alteration on proliferation may be masked by EGFR overexpression or other genetic alterations. These are speculative explanations for how CXCL16 appears not to affect proliferation of breast cancer cells.

In addition, since CXCL16 exists both as a transmembrane form and a soluble form, and the former could be cleavaged off into the soluble form, it is difficult to distinguish precisely which form is being studied in which cancer experiments. In fact, it has been reported that a molecule ADAM10 mediates the conversion of transmembrane CXCL16 into soluble CXCL16 dynamically [4, 5]. Therefore, in this study we defined CXCL16 as “cellular CXCL16”, which exists predominantly in the cell membrane. To characterize it, we employed flow cytometry to quantify CXCL16 localized on cell surfaces in living cancer cells. We report high cell surface expression of CXCL16 on living cells, in proportion to total CXCL16 expression by cells. Our data suggest that the transmembrane CXCL16 is involved in breast cancer cells with important clinical significance.

In summary, the current study demonstrated that cellular CXCL16 expression was negatively correlated with the migration and invasion of breast cancer cells. Moreover, overexpression or downregulation of CXCL16 could inhibit or facilitate these malignant behaviors.
